# Bubonic plague: can the size of buboes be accurately and consistently measured with a digital calliper?

**DOI:** 10.1186/s13063-023-07835-7

**Published:** 2023-12-19

**Authors:** Josephine Bourner, Ravaka Randriamparany, Tsinjo Fehizoro Rasoanaivo, Emmanuelle Denis, Rindra Vatosoa Randremanana, Michel Vaillant, Alex Paddy Salam, Bronner P. Gonçalves, Piero Olliaro

**Affiliations:** 1 Pandemic Sciences Institute, University of Oxford, Oxford, UK; 2 Institut Pasteur de Madagascar, Antananarivo, Madagascar; 3 Luxembourg Institute of Health, Strassen, Luxembourg

## Abstract

**Introduction:**

Conducting clinical research on treatments for emerging infectious diseases is often complicated by methodological challenges, such as the identification of appropriate outcome measures to assess treatment response and the lack of validated instruments available to measure patient outcomes. In bubonic plague, some studies have assessed bubo size as an indicator of treatment success, a measure widely assumed to be indicative of recovery. Evaluating this outcome however is challenging as there is no validated method for measuring bubo size. The aim of this study is to assess the accuracy and inter- and intra-rater agreement of artificial bubo measurements using a digital calliper to understand whether a calliper is an appropriate measurement instrument to assess this outcome.

**Methods:**

Study technicians measured 14 artificial buboes made from silicone overlaid with artificial silicone skin sheets over the course of two training sessions. Each artificial bubo was measured by each study technician once per training session, following a Standard Operating Procedure. The objectives of this study are to (i) evaluate the accuracy of individual measurements against the true size of the artificial bubo when using a digital calliper, (ii) understand whether the characteristics of the artificial bubo influence measurement accuracy and (iii) evaluate inter- and intra-rater measurement agreement.

**Results:**

In total, 14 artificial buboes ranging from 52.7 to 121.6 mm in size were measured by 57 raters, generating 698 measurements recorded across two training sessions. Raters generally over-estimated the size of the artificial bubo. The median percentage difference between the measured and actual bubo size was 13%. Measurement accuracy and intra-rater agreement decreased as the size of the bubo decreased. Three quarters of all measurements had a maximum of 25% difference from another measurement of the same artificial bubo. Inter-rater agreement did not vary with density, size or presence of oedema of the artificial bubo.

**Conclusions:**

The results of this study demonstrate the challenges for both individual and multiple raters to repeatedly generate consistent and accurate measurements of the same artificial buboes with a digital calliper.

**Supplementary Information:**

The online version contains supplementary material available at 10.1186/s13063-023-07835-7.

## Introduction

Bubonic plague, like many other neglected infectious diseases of poverty, disproportionately affects some of the most economically vulnerable communities in the world. Combined, reports from Madagascar and the Democratic Republic of Congo account for approximately 98% of the global cases of plague [1]—although cases are likely underreported due to the disease predominantly arising in remote rural areas and the non-specific presentation of symptoms making recognition of plague challenging [2, 3].

Recommended to treat bubonic plague are antibiotics in the aminoglycoside, fluoroquinolone and tetracycline families, among others [4]. However, as a result of historical under-investment in research, exacerbated by lack of commercial interest, there is an absence of clinical evidence to support the different antibiotic regimens that are currently in use. Further, global drug shortages [5] and reports of antimicrobial resistance of *Yersinia pestis*—the gram-negative bacterium that causes plague in humans—to streptomycin [6], which has long been prioritised as the first-line treatment regimen for plague, means identifying alternative treatment regimens is now critical. There are other drugs that are used to treat bubonic plague, such as gentamicin, ciprofloxacin and doxycycline, all of which have been incorporated into treatment guidelines and clinical practice and have received approval under the U.S. Food and Drug Administration’s ‘Animal Rule’ [7], but there is no robust trial data in humans documenting their efficacy. The results of only two clinical trials in humans evaluating the safety and efficacy of plague therapeutics have been published to date [8, 9], and a third trial is currently ongoing [10]. Unfortunately, both studies failed to generate conclusive evidence for the drugs evaluated under their protocols, partly because of logistical and methodological challenges.

More generally, substantial methodological challenges exist when conducting clinical research on bubonic plague, for which the sporadic case numbers would result in low enrolment to trials and, in the event that mortality is used as a primary endpoint, the numbers of mortality events would be too low for significant treatment effects to be detected between arms [11]. Other endpoints to demonstrate therapeutic efficacy are therefore needed. Importantly, alternative endpoints must be clinically meaningful to patients and observed with sufficient frequency that treatment effects can be detected and reliably measured. However, this proves challenging for plague—as well as other epidemic-prone infectious diseases such as Lassa fever and monkeypox [12]—as the clinical evolution of the disease and patient outcomes have historically been poorly documented, making it challenging to identify outcome measures that adequately capture how a patient feels, functions or survives [13]. Of the three clinical trials that have taken place in recent years, two employ a composite primary endpoint that evaluates several clinically meaningful outcomes to assess treatment response, such as survival, resolution of fever, reduction in size or resolution of the bubo [8, 10].

Evaluation of the size of the bubo is an outcome that is sometimes reported in clinical studies of bubonic plague and has been used to detect clinical recovery in a trial setting [10]. The bubo is a typical clinical feature of plague, with one bubo being typically detected in approximately 96% of cases at presentation, but has been poorly defined in scientific literature [2]. Across 91 clinical studies published in peer-reviewed journals between 1902 and 2021, 18 recorded the size of the bubo on at least one occasion [2]. Of the three clinical trials of bubonic plague that have been initiated to date [8–10], two measured bubo size for at least one timepoint [8, 10]. However, using these data in a clinical and research settings—particularly if it is used to evaluate an endpoint in a clinical trial—is complicated by the fact that there is no validated method for measuring bubo size.

Few studies in the existing literature report the bubo measurement method. While several options for measuring buboes may be considered, including using ultrasound, CT imaging [14] and digital calliper [10], the calliper may be the most practical option in plague-endemic settings, due to its low cost, accessibility and no requirement for specialist training. There are no data however informing the accuracy or inter- and intra-rater agreement of the digital calliper approach. There is therefore uncertainty around the scale of the potential measurement error that could hinder the accurate assessment of changes in bubo size over time—which poses a substantial risk where bubo evolution is used for the evaluation of clinical trial endpoints.

The aim of this study was to assess the accuracy and inter- and intra-rater agreement of artificial bubo measurements by clinical study technicians (*Téchniciens d’Etude Clinique*, or TECs) working on the IMASOY trial using a digital calliper. The TECs involved in this exercise were those responsible for measuring buboes in patients enrolled in the IMASOY trial at peripheral health centres in Madagascar.

The results presented in this manuscript have been reported according to the Guidelines for Reporting Reliability and Agreement Studies (GRRAS) [15].

## Methodology

### Preparation of artificial buboes

**Fig. 1:**
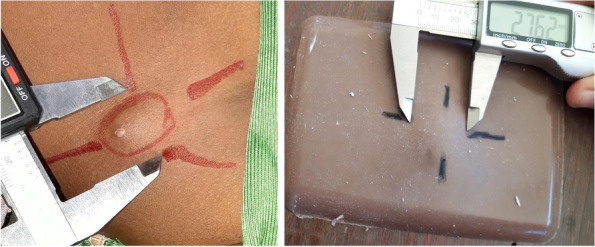
Measurement of a bubo encountered in a clinical setting versus an artificial bubo

The study was conducted using 14 artificial silicone buboes. Bubo sizes were selected following review of data reported to Institut Pasteur de Madagascar (IPM) by clinicians with experience treating plague, from which it was estimated that approximately three quarters of buboes have a long axis of approximately 35–55 mm range. To mimic challenging conditions encountered in clinical practice (Fig. 1), whereby buboes are poorly delimited and embedded in surrounding oedema and inflammation, the 14 artificial buboes were made in seven different sizes with a range in the long axis of 29.8–81.8 mm, with a hard or soft silicone bubo and with or without an additional silicone layer representing oedema (Table 1). The same set of artificial buboes was used in each training session.

**Table 1:** Dimensions and characteristics of artificial buboes

Bubo ID	Short-axis measurement (mm)	Long-axis measurement (mm)	Total size (long axis + short axis) (mm)	Characteristic	
				**Density**	**Presence of oedema**
1	39.8	81.8	121.6	Hard	No
2	39.8	81.8	121.6	Soft	Yes
3	50.2	70.1	120.3	Hard	No
4	50.2	70.1	120.3	Soft	Yes
5	50.2	70.1	120.3	Soft	No
6	32.7	62.0	94.7	Hard	Yes
7	32.7	62.0	94.7	Hard	No
8	31.5	46.3	77.8	Hard	No
9	31.5	46.3	77.8	Soft	Yes
10	31.5	46.3	77.8	Soft	No
11	26.8	38.1	64.9	Hard	No
12	26.8	38.1	64.9	Soft	No
13	22.9	29.8	52.7	Hard	No
14	22.9	29.8	52.7	Soft	No

**Fig. 2:**
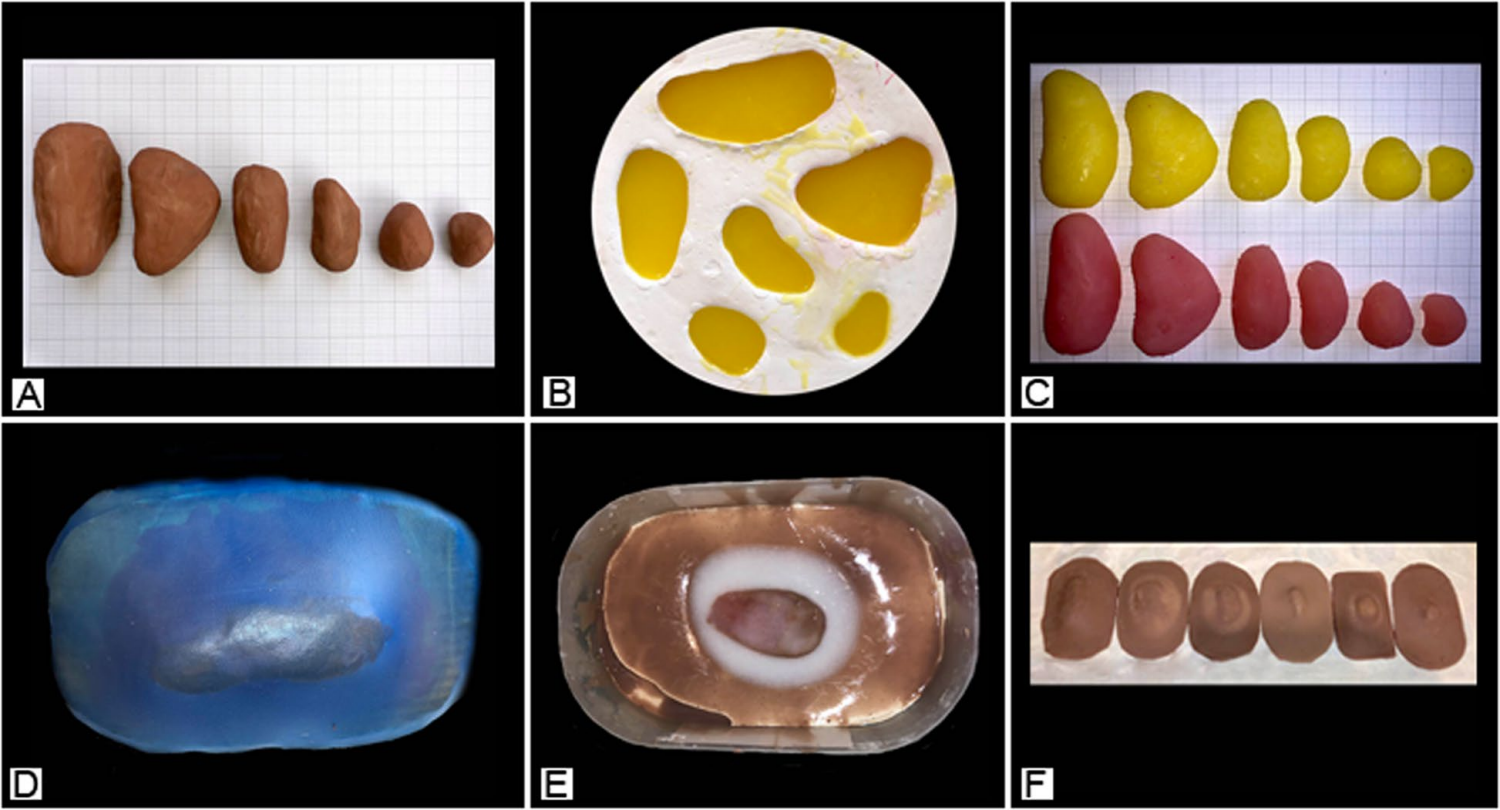
Artificial bubo preparation. **A** Standardised clay buboes. **B** Casting the buboes. **C** Set of soft (yellow) and hard (pink) buboes. **D** The outer bubo mould. **E** Casting a bubo in the outer mould with a layer of Soma Foama™ 15 to create the oedema effect. **F** A competed set of artificial buboes

The artificial buboes were created by first preparing a set of standardised clay buboes with the axes as described in Table 1. A negative volume mould was then prepared using Crystacal R Casting Plaster and the soft and hard buboes were cast using Ecoflex™ 00–10 and Ecoflex™ 00–20 (Smooth-On, Inc. Macungie, PA). A second negative volume mould was created by spreading a thin layer of Dragon Skin FX‑ Pro™ (Smooth-On, Inc. Macungie, PA) over a skin-textured vinyl sheet. The resulting skin-textured latex sheet was overlaid on a clay form and this was used to make the outer bubo Crystacal R Casting Plaster mould. The outer bubo mould was coated in Dragon Skin and, once the initial layer has cured, a bubo was placed in the cavity and overlaid with more Dragon Skin. The oedema effect was achieved by adding a layer of Soma Foama™ 15 around the bubo and allowing it to cure before overlaying with Dragon Skin (Fig. 2).

### Measurement process

During the course of two training sessions taking place in August 2020 and August 2021, raters were asked to measure each artificial bubo once using a digital calliper and record the long- and short-axis measurements (in mm) on a standardised data collection form, which was then transferred into a Microsoft Access database. Measurements were made individually and all observers remained blind to the measurements of others.

**Fig. 3:**
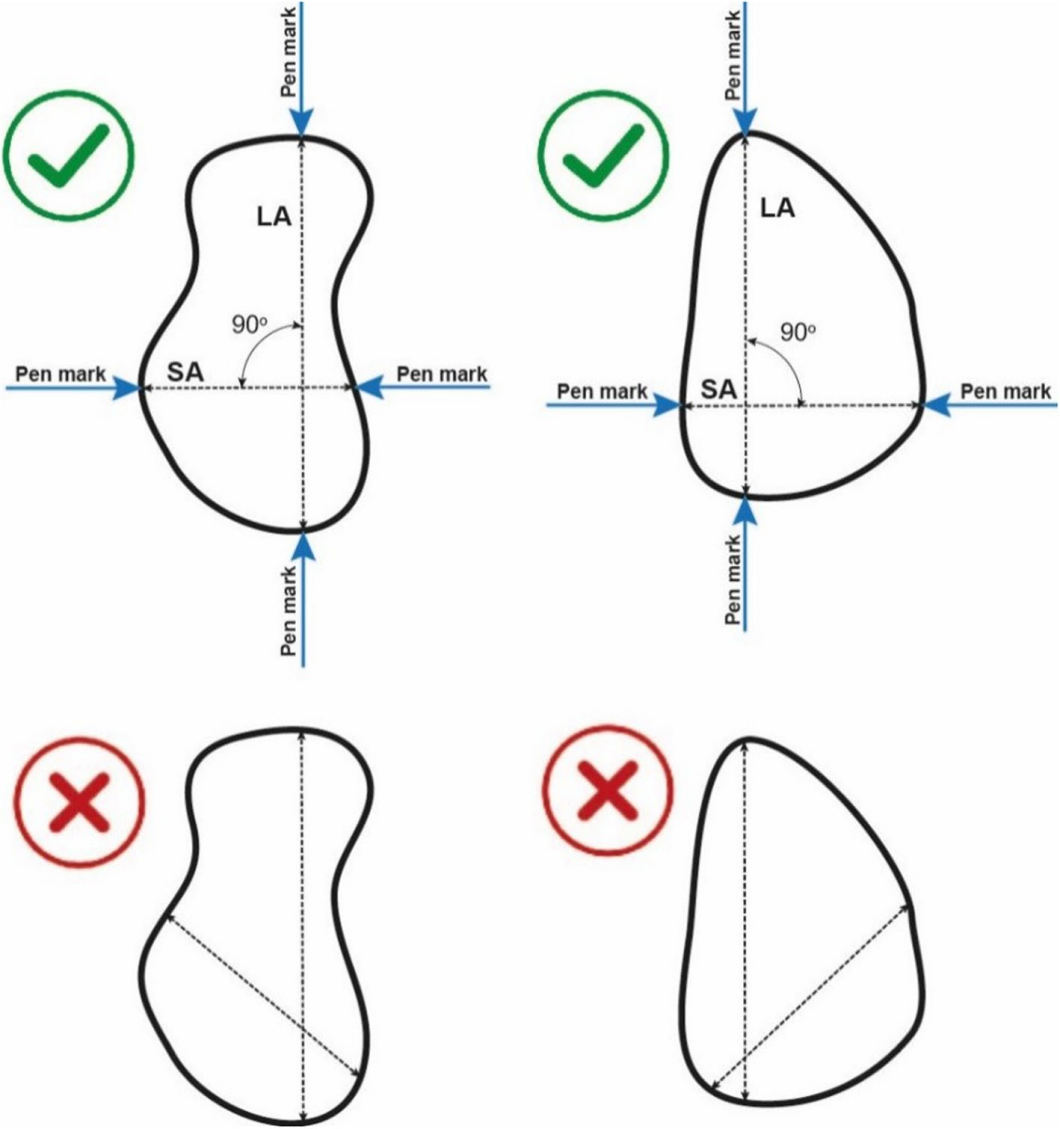
Bubo measurement method

Artificial buboes were measured as described in the IMASOY trial protocol and relevant SOP (S1 Text): the bubo is palpated to understand its shape and the location of the long and short axes. The long axis is first identified and the short axis is defined as the longest measurement perpendicular to the long axis. The long axis is then measured by placing an eyeliner pencil a couple of centimetres above and below the identified axis and drawing a line as if to bisect the bubo, stopping when the pencil meets the margin of the swelling (Fig. 3). The same actions are repeated for the short axis. The space between the pencil marks on the long and short axes are then measured using a digital calliper and recorded in mm.

### Objectives

Three analyses were undertaken to assess measurement accuracy, and inter- and intra-rater agreement. Accuracy determines how closely an individual rater can record a measurement of the artificial bubo to its true size. This is measured as the percentage difference and absolute difference in millimeters of the measured size to the true size. Intra-rater agreement measures the extent to which an individual rater can generate similar repeated measurements of the same bubo; inter-rater agreement is the extent to which multiple raters can record similar measurements of the same object.

In order to understand how accurately buboes may be measured in a real-world setting, the primary objectives of this study were to (i) evaluate the accuracy of individual measurements against the true size of the artificial bubo when using a digital calliper and (ii) understand whether the characteristics of the artificial bubo influence measurement accuracy.

As multiple measurements may be taken by either a single rater or multiple raters in the context of clinical or research study follow-up, it is therefore important to understand the reproducibility of measurements within and between individual raters. Furthermore, this study aims to understand the implications of using a digital calliper in a real-world setting, where the true size of the bubo may not be known. The variation between multiple measurements of the same bubo would therefore reveal the extent of the measurement error that may exist in the absence of more sophisticated technology to measurement bubo size (such as ultrasound).

### Participants and sampling method

The raters were the clinical study technicians (TECs), who have a nursing qualification, employed by the IMASOY trial to support trial sites with study activities and who attended trial training sessions in August 2020 and August 2021. Prior to this, the TECs had limited or no experience measuring buboes in a clinical setting using a digital calliper.

The TECs are responsible for the measurement of buboes in patients enrolled in the IMASOY trial and receive annual trial-specific training in August each year, which includes a session dedicated to the bubo measurement technique using artificial buboes.

**Fig. 4:**
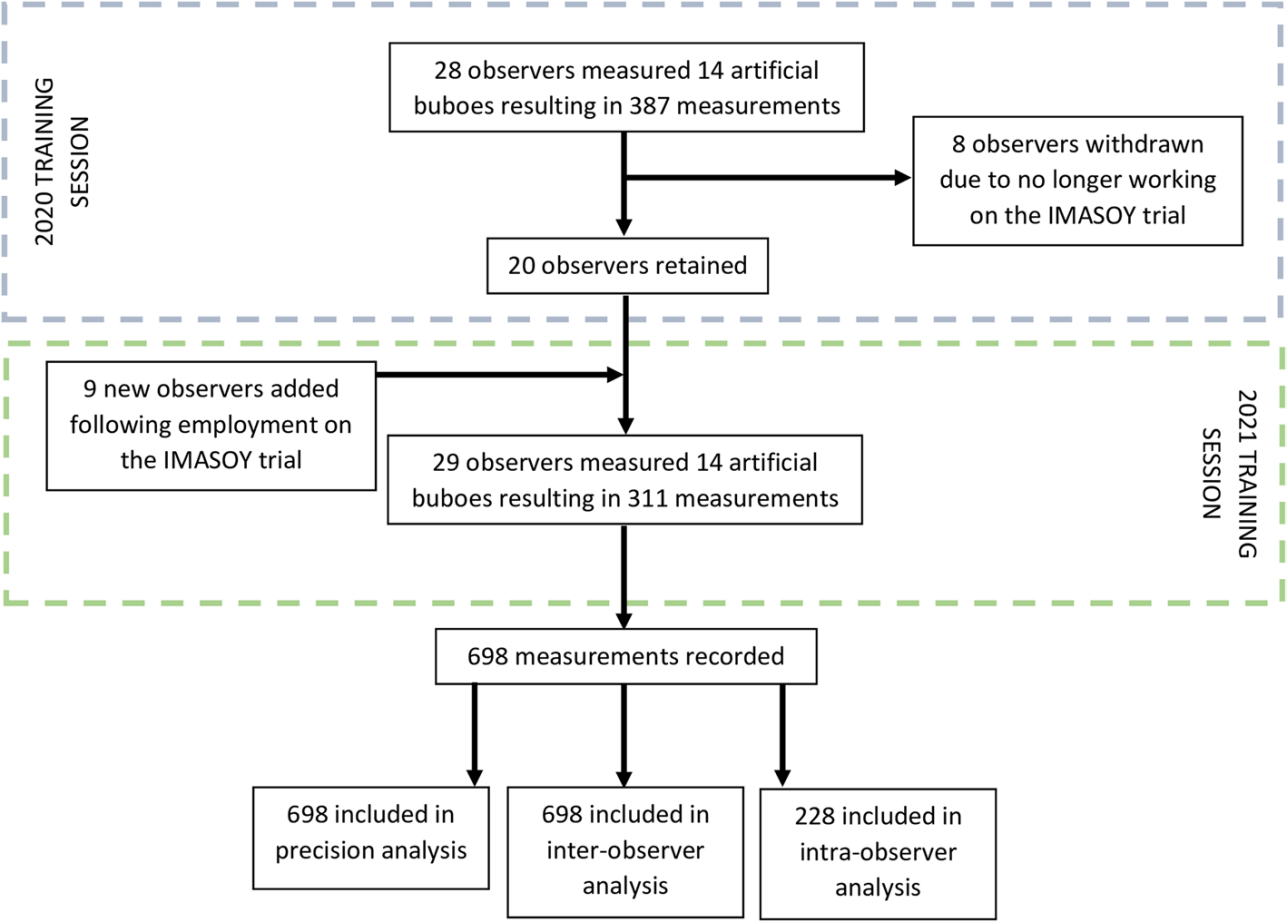
Flowchart of rater participation and recorded measurements per training session

As this study was integrated in to the annual training session of the ongoing IMASOY clinical trial, the sample was chosen through convenience. In August 2020, 28 TECs participated in the training and in August 2021 29 TECs participated in the training (Fig. 4). There were 20 TECs who participated in both training sessions. Data from both training sessions are included in this analysis.

### Analysis

The following analyses were carried out using RStudio v. 1.3.1093 and in Microsoft Excel. Where *p*-values are reported, we have used a significance level of 0.05.

### Accuracy

An analysis was conducted to evaluate the accuracy of the measured artificial bubo size compared to the true size (Table 1). The measured difference (in mm) and percentage difference of the measurement from the true artificial bubo size was calculated for each recorded measurement.

Measurement accuracy was evaluated by comparing the true size of the artificial bubo with the percentage difference of the measurements, using the median percentage difference of all measurements and by summarising the number of measurements that fell within intervals increasing in 5% increments from the true size. Here, as in the other analyses across the paper, the intervals of 5 and 25% have been selected to summarise the number of measurements made within these boundaries (although a full break-down of the number of measurements made within each interval can be found in S5 Table).

To evaluate the impact of the bubo characteristics (density, size and presence of oedema) on the percentage difference between the measurements and the artificial buboes’ true size, a linear mixed effects regression model with a random effect for rater ID was fitted with a fixed effect for density (hard, soft), oedema (present, absent), year (2020, 2021) and size (five dummy variables to allow for the six sizes). We present the regression coefficient to demonstrate the mean response of the measurement difference when there is a change in the corresponding characteristic, along with the 95% confidence interval (CI).

Scatterplots are also presented to show the percentage difference between each measurement and the true size of the artificial bubo according to size, presence of oedema and density, with a regression line and 95% CI.

### Intra-rater agreement

Intra-rater analyses are reported across all artificial bubo measurements and by characteristic (density, size and presence of oedema).

To evaluate the extent to which an individual can record similar repeated measurements of the same artificial bubo (the intra-rater agreement) 1 year apart, we present the number of second measurements made by each rater that were recorded within 5% intervals of their first measurement of the same artificial bubo.

To assess the effect of the bubo characteristics on the percentage difference between the raters’ second measurement compared to their first measurement, a linear regression was conducted using a mixed effects model. Results are presented in a scatterplot and Supplementary table.

We also present the mean absolute difference and standard deviation between the raters’ first and second measurements.

Bland–Altman plots were generated by plotting each rater’s average measurement (mm) (measurement 1 + measurement 2/2) against the absolute difference (mm) between the measurements with lines depicting the mean difference and Limits of Agreement (LoA) (mean difference ± 1.96 SD).

As the artificial buboes were measured once by each rater during each training session, analysis of intra-rater measurements was conducted using data only from raters who participated in both training sessions (*n* = 20); data from those who participated in only one training session were excluded from this analysis.

### Inter-rater agreement

To evaluate the extent to which an individual can record similar measurements to those of other raters (the inter-rater agreement), we present the number of measurements that were recorded within 5% intervals of other raters’ measurements of the same artificial bubo.

## Results

In total, 698 measurements were recorded across the two training sessions by 57 raters overall, of whom 20 recorded measurements in both training sessions (S2 Data). The median number of artificial buboes measured per rater per year was 12 (range 5 to 14). Of the 57 raters, 29 (51%) measured all 14 artificial buboes.

### Accuracy

The overall median percentage difference of all artificial bubo measurements compared to the reference bubo size was 13%, with a range of 0 to 171% (Table 2). There were 179 (26%) measurements made within 5% of the reference size, and 508 (73%) within 25% of the reference size.

**Table 2:** Summary of the total number of measurements, median absolute difference and median % difference (and range) from the reference size of measurements recorded per artificial bubo characteristic

Artificial bubo characteristic	*N* recorded measurements	Median absolute difference (mm)	Median absolute % difference	Difference range (mm)	% difference range
Overall	698	12.0	13%	0.01 to 89.92	0 to 171%
Hard	349	12.3	13%	0.01 to 89.92	0 to 171%
Soft	349	11.9	12%	0.01 to 76.41	0 to 145%
Oedema	213	10.3	10%	0.07 to 72.95	0 to 94%
No oedema	485	12.6	15%	0.01 to 89.92	0 to 171%
Size = 121.6	108	10.7	9%	0.32 to 73.04	0 to 60%
Size = 120.3	160	9.7	8%	0.14 to 56.19	0 to 47%
Size = 94.7	88	9.7	10%	0.07 to 68.19	0 to 72%
Size = 77.8	150	12.1	16%	0.26 to 82.51	0 to 106%
Size = 64.9	86	11.8	18%	0.01 to 46.84	0 to 72%
Size = 52.7	106	17.6	28%	0.01 to 89.92	0 to 171%
Year: 2020	387	10.69	10%	0.01 to 89.92	0 to 171%
Year: 2021	311	15.75	17%	0.01 to 82.51	0 to 139%

More raters over-estimated the size of the artificial bubo than under-estimated (S3 Figure). Of the recorded measurements, 507 (74%) were recorded larger than the true artificial bubo size while 180 (26%) were recorded smaller than the true artificial bubo size. Of the individual raters, 47 (82%) more frequently over-measured artificial buboes compared to their true size.

**Fig. 5:**
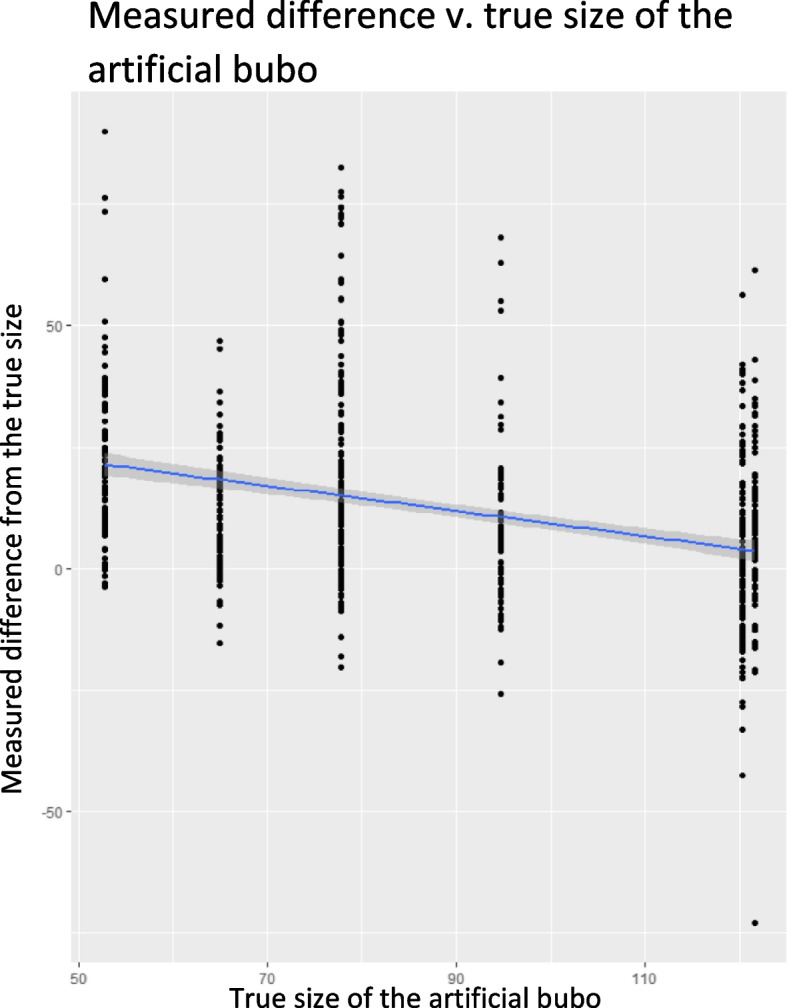
Scatterplot of percentage difference of each measurement from the true size plotted against the true size for per characteristic (size and presence of oedema)

Size and year of training had a significant effect on measurement accuracy (S5 Table – A; S5 Table – B). Measurement error significantly decreased as the size of the artificial buboes increased (Fig. 5 and S5 Table A). The median measurement error of the three largest artificial buboes (measuring 121.6 mm, 120.3 mm, 94.7 mm) ranged from 8 to 10% (Table 2). The largest measurement error was seen for the smallest artificial bubo with a median measurement error of 28% (Table 2).

The year in which the measurements were conducted also influenced measurement accuracy. Artificial buboes were more accurately measured in 2020 than in 2021 (Table 2), where there was statistically significant evidence of over-estimation of the true size (S5 Table –A).

Neither the presence of oedema nor density had a significant impact on measurement accuracy.

### Intra-rater agreement

**Fig. 6:**
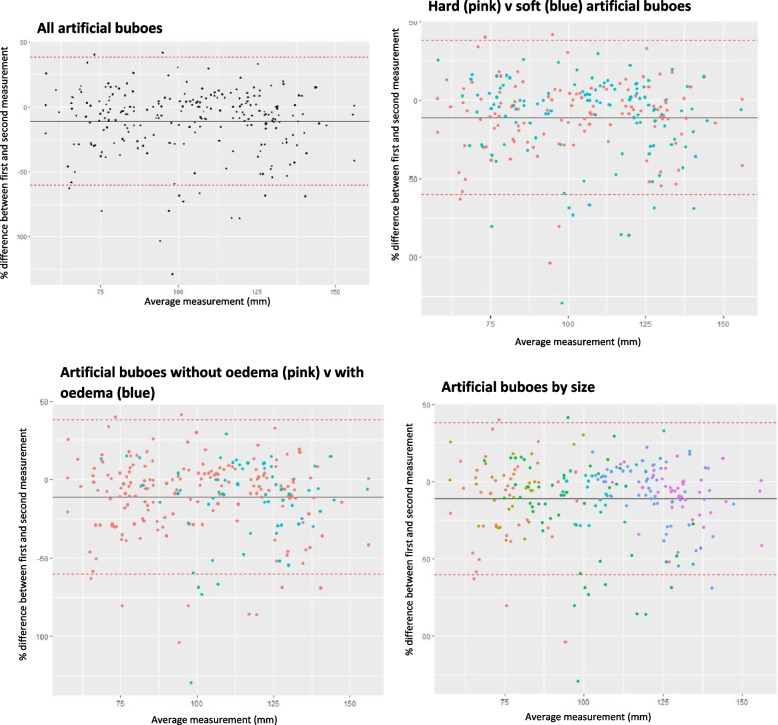
Bland–Altman plots showing the intra-rater agreement of the average measurement and percentage difference between measurements by artificial bubo characteristic

Twenty raters measured the artificial buboes in two training sessions generating 239 pairs of measurements. The median percentage difference between the raters’ first and second measurement of the same artificial bubo was 11%, with a range of 0 to 129% (S5 Table – C; Fig. 6). The median absolute difference between the first and second measurement was 11.84 mm with a range of 0.03 to 76.87 mm.

On average, each rater measured 12/14 (86%) artificial buboes twice, of which 3 (25%) of the second measurements were within 5% of the first measurement and 9 (75%) were within 25% (S5 Table – D).

Ninety-five percent of all second measurements of hard artificial buboes were recorded within 55% of the first measurement and soft artificial buboes were measured within 70% (S5 Table – E). Ninety-five per cent (95%) of all second measurements of artificial buboes with oedema were recorded within 60% of the first measurement, and artificial buboes without oedema were measured within 55% (S5 Table – E).

**Fig. 7:**
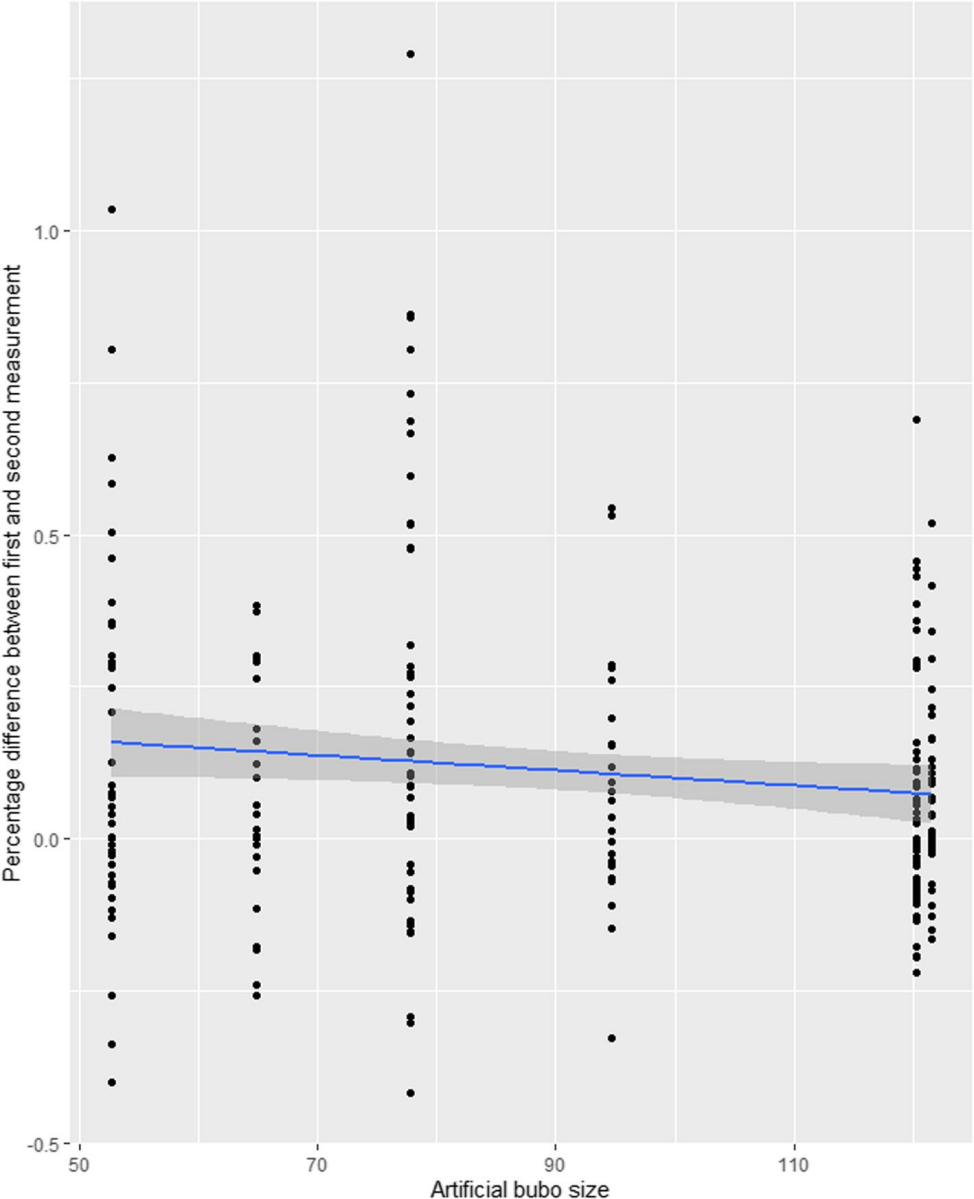
Scatterplot of the percentage difference between the first and second measurements of the same artificial bubo plotted against the true size of the artificial bubo

The linear regression demonstrated that size had a statistically significant effect on the agreement between the first and second measurement of the same artificial bubo by the same rater (S5 Table – F). Intra-rater agreement increased as the size of the artificial bubo increased (Fig. 7). Neither density nor presence of oedema had a statistically significant effect on intra-rater agreement.

### Inter-rater agreement

Overall, the median standard deviation between measurements of the same artificial bubo by different raters was 17.1 mm, with a range of 10.3 to 23.0 mm. Seventy-five percent (75%) of the raters were able to record a measurement within 25% of another rater who measured the same artificial bubo, and approximately one fifth could record a measurement within 5% of another rater (S5 Table – G).

None of the bubo characteristics (density, size or presence of oedema) had a statistically significant impact on the raters’ ability to measure the artificial buboes within 25% of other raters’ measurements of the same artificial bubo.

## Discussion

This study aimed to evaluate whether a digital calliper can be used to accurately and consistently measure artificial buboes, with the goal of determining its suitability for use in a clinical research setting. This study also considered the implications of wider use as a measurement tool in the context of assessing reduction in bubo size as a component of response to treatment.

Overall, almost three quarters of raters were able to measure the artificial buboes within 25% of their true size, with a median measurement difference of 13%. However, this means that about one-quarter of all measurements were greater than 25% of the artificial buboes’ true size and, in some cases, substantial measurement error—up to 170%—was observed.

Measurement accuracy was impacted by the year in which the measurement was made and size of the artificial bubo, with larger artificial buboes being measured most accurately, and an increase in measurement error being seen as artificial bubo size decreases. Due to the tendency for the size of smaller artificial buboes to be over-estimated, where reduction in bubo size is used as an indicator of recovery, there is a risk of underestimating treatment effect size and not detecting changes in size of smaller buboes.

While this enhanced risk could be mitigated by having the same rater repeat measurements throughout the course of treatment, a substantial proportion of all measurements—approximately one-third—were recorded more than 25% smaller or larger than the original measurement and, once again, size was seen to modulate agreement with larger artificial buboes generating greater intra-rater agreement. This again reinforces the risk of inaccurate estimation of treatment effect where bubo size is used as an indicator recovery as individual rater’s measurements show a greater risk of divergence with decreasing bubo size.

The artificial buboes were originally developed as a training tool to overcome the logistical challenges of training a high number of staff to measure buboes ahead of the transmission season of bubonic plague. A limitation of this study is that data were collected using artificial buboes—for which each of the permutations of size, density and presence of oedema were not present across the various sizes—rather than from patients diagnosed with bubonic plague. Due to limited existing literature characterising the bubo, it is unclear how accurately the artificial buboes mimic all possible forms and permutations that may be encountered in a clinical setting. There may be added complications to consider when measuring buboes in patients; buboes may be located in challenging anatomical locations and pain associated with the bubo may prevent accurate measurement. The inferences made in this analysis should therefore be viewed with caution.

The study was not designed to determine the likely prevalence of incorrect determination of a pre-specified reduction in size to inform evaluation of treatment response. The results of this study do however provide parameter data and values to inform the design and conduct of a simulation study that could estimate (i) how often a pre-specified percentage reduction in bubo size would be correctly classified under different assumptions about measurement error and (ii) the implications for estimation of the treatment effect between arms and the likelihood of drawing an incorrect conclusion in a randomised controlled trial.

## Conclusions

The results of this study therefore demonstrate the challenges for both individual and multiple raters to repeatedly generate consistent and accurate measurements of the same artificial buboes with a digital calliper.

This study also highlights the challenges surrounding the selection of clinically relevant and measurable endpoints in a clinical trial for difficult to study infectious diseases—particularly when mortality cannot be used as a viable endpoint and there is a lack of data on the natural history of the disease to support alternatives. A recent systematic review has highlighted the deficiencies in our current understanding of the bubo and its relationship with treatment outcomes. This, coupled with the uncertainty around a suitable measurement method, indicates that more work is needed to establish clinically relevant indicators of recovery from plague, the role of the bubo in the disease and reliable measurement methods of plague characteristics.

### Supplementary Information

**Additional file 1: S1 Text.** Bubo measurement SOP.

**Additional file 2: S2 Data.**Full raw data.

**Additional file 3: S3 Fig.** Histograms showing the distribution of measurements in percentage difference from the true size of each artificial bubo.

**Additional file 4: S4 Fig.** Box plot showing the percentage difference between the true size of the artificial bubo and the measured size per characteristic.

**Additional file 5: S5 Table.** Summary of measurements.

**Additional file 6. **GRAAS checklist.

## Data Availability

All data generated or analysed during this study are included in this published article (see S2 Data).

## Abbreviations

IPM: Institut Pasteur de Madagascar
LoA: Limits of Agreement
TEC: Téchnicien d’Etude Clinique
