# Supplementary material for: Bubonic plague: can the size of buboes be accurately and consistently measured with a digital calliper?
Source: Trials. 2023 Dec 19;24:815. doi: 10.1186/s13063-023-07835-7 (PMC10729355; doi:10.1186/s13063-023-07835-7)
Supplement: Supplementary file 1 — Additional file 1: S1 Text. Bubo measurement SOP. [file 13063_2023_7835_MOESM1_ESM.pdf]

1. Objective

The objective of this standard operating procedure (SOP) is to describe the steps that should be followed to perform bubo measurements using a digital calliper.

2. Application

This SOP describes how to perform, record and photograph the measurements of buboes on days 1, 4, 11 and 21 after inclusion for patients enrolled in the IMASOY trial.

3. Responsibilities

The study technician (TEC) is responsible for :

- Correctly positioning the patient to take the bubo measurement in accordance with the study schedule
- Recording the measurement on the paper CRF
- Taking a bubo of the measurement

4. Equipment

4.1. Digital calliper

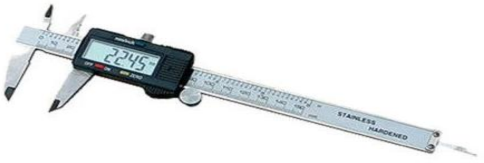

4.2. Paper towels

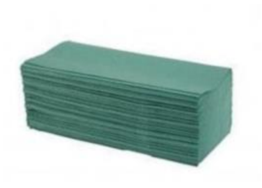

4.3. Eye-liner

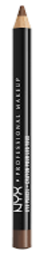

4.4. Make-up remover

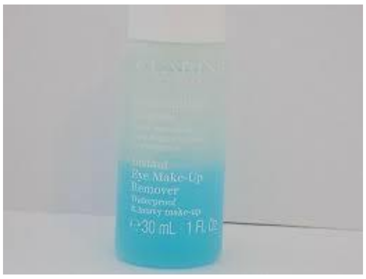

4.5. Cotton balls

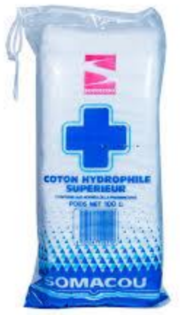

4.6. Gloves

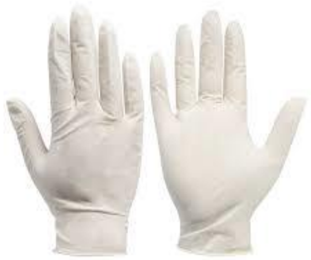

5. Procedure

5.1. Positioning the patient

Before taking the measurement, patient should be positioned in the following way, depending on the location of their bubo:

a. **Cervical bubo:** The patient should be seated with the neck slightly bent forward
b. **Axillary bubo:** The patient should be lying down, with their arm stretched over and around to the other side of the head
c. **Inguinal bubo:** The patient must be in the supine position with the hips stretched or slightly flexed (extra-rotated)

It should be noted that if it is not possible to place the patient in the positions described above, place the patient in a position that allows the TEC to hold the patient comfortably during the bubo measurement, but also to have adequate access to the bubo for the measurement.

5.2. Taking the measurments with the calliper

After the patient has been correctly positioned (see section 5.1 above), the TEC will measure the bubo following the instructions below.

- After donning gloves, the TEC will gently palpate the bubo to feel its shape and the location of the long and short axes. As the TEC palpates the bubo, they should also ensure that they have identified any oedema that may exist around the bubo. Remember, we want to measure the bubo - not the oedema.
- The TEC first identifies the long axis of the bubo. They then place the pencil gently on the patient's skin a few centimetres above the longest axis and draws a straight line as if to cut the bubo in half along its long axis, but stopping when the pencil meets the edge of the swelling.

Apply only enough pressure to leave a mark on the pencil and stop drawing the line when the pencil stops naturally at the edge of the swelling. Do not draw any lines on the swelling and do not encircle the swelling.
The TEC then places the pencil on the patient's skin a few centimetres below the major axis and draws a straight line towards the bubo which aims to join the other line, but stops again when the pencil meets the edge of the swelling.

- The TEC repeats the same steps for the measurement of the short axis.

Note that the short axis is defined as the longest measurement perpendicular to the longest axis (see figure 1).

Figure 1 – Positioning of pencil marks in relation to the bubo

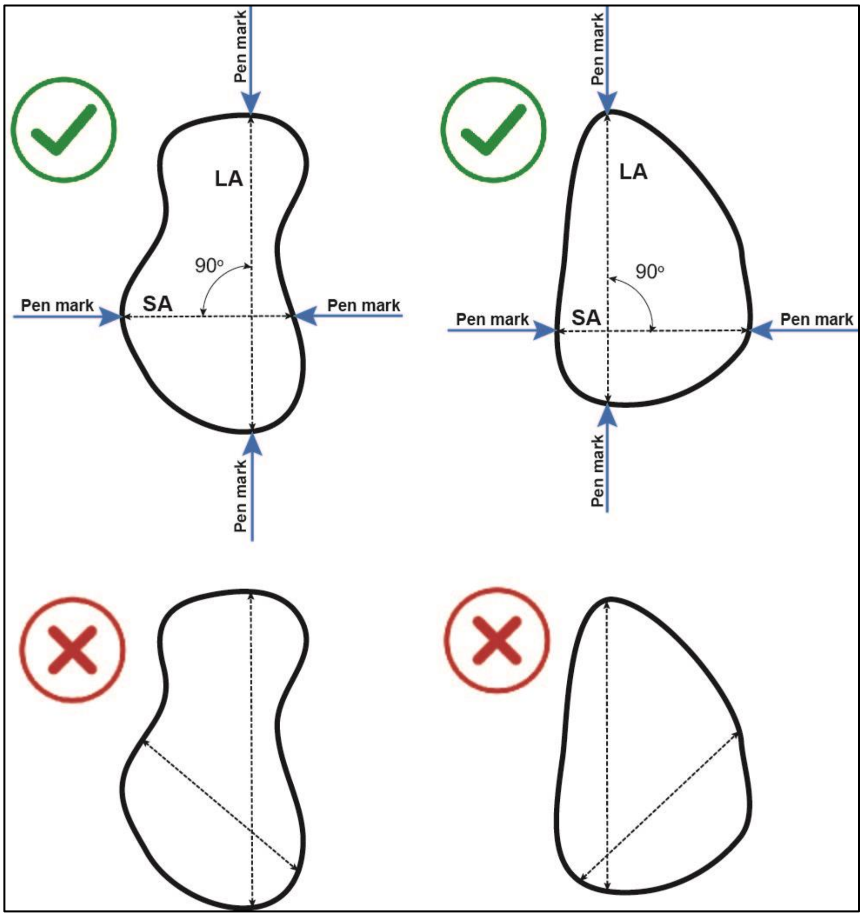

- The TEC then takes the digital caliper and adjusts its outer jaws to measure the space between the pencil marks on the long axis and the short axis.
- The measurement shown on the digital caliper screen must be recorded on the paper CRF. The two TECs must agree on the final measurement to be recorded on the CRF.
- The TEC takes a photo of the bubon and the measurement shown on the caliper screen. The measurement on the screen must be legible on the photo: the face of the screen is placed flat towards the camera and not at an angle (see section 5.8).

- The eyeliner marks should then be removed from the patient's skin using cotton wool and make-up remover.

5.3. Photos of the measurement

All photos will be taken using the tablet or another device of the same quality; make sure the camera is parallel to the surface of the body, ideally about 15-20 centimetres above the bubo. Photos should be taken from both the long and short axes.

When taking photographs of caliper measurements, the pencil marks on the skin and the caliper screen must be clearly visible. When the caliper screen is positioned along the pencil lines, the screen must also be positioned so that the marks are flat towards the camera and not at an angle.

After taking the photo and before removing the pencil marks from the patient's skin, the TEC should check that the photo is of sufficient quality for the measurement to be easily read (even if the photo has to be enlarged to read the measurement, the quality must not be lost). If the photo is not of sufficient quality, further photos should be taken until the TEC is satisfied with the quality.

All photos must be immediately recorded on the tablet or alternative device and labelled with the patient's identification number, the day of the measurement visit and the bubo number (which can be found on the paper CRF under "bubo site"):

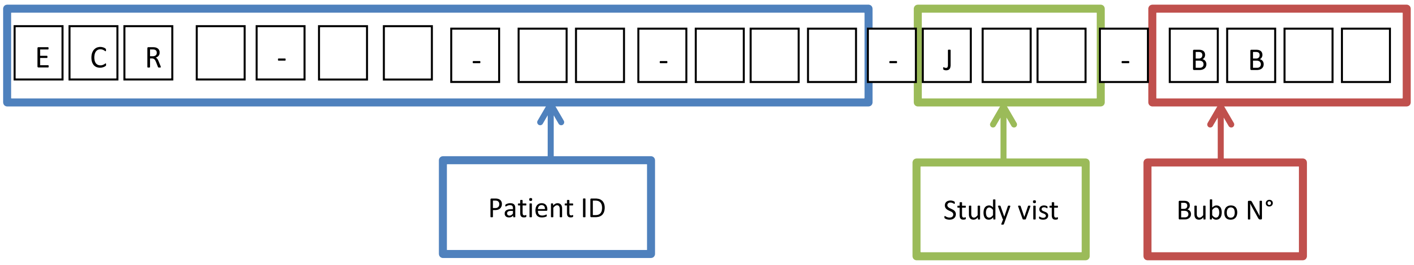

5.4. Data entry

Each bubo must be given an identifier:

- If the patient has only one bubo, he or she will be given the identifier "BB-01".
- If the patient has more than one bubo, each bubo will be numbered sequentially, i.e. "BB-01", "BB-02", "BB-03".

It does not matter how each bubo is numbered, as long as the number corresponding to each bubo is remembered. The number assigned to each bubo at D1 will be the number it will have at each subsequent visit.

If the patient has more than one bubo, use the image in Appendix 1 to help remember which number is allocated to each bubo by marking the location of each bubo and its identification number.

The bubo size measurement section of the CRF should be completed for each patient.

When completing the pCRF, particular attention was paid to the following elements:

- Location of bubo
- Axis of measurement

If buboes disappear during the course of the study, information about the bubo should still be recorded on the CRF, including the measurement as 0.00 mm x 0.00 mm.
