# Supplementary figures and images for: Bubonic plague: can the size of buboes be accurately and consistently measured with a digital calliper?
Source: Trials. 2023 Dec 19;24:815. doi: 10.1186/s13063-023-07835-7 (PMC10729355; doi:10.1186/s13063-023-07835-7)

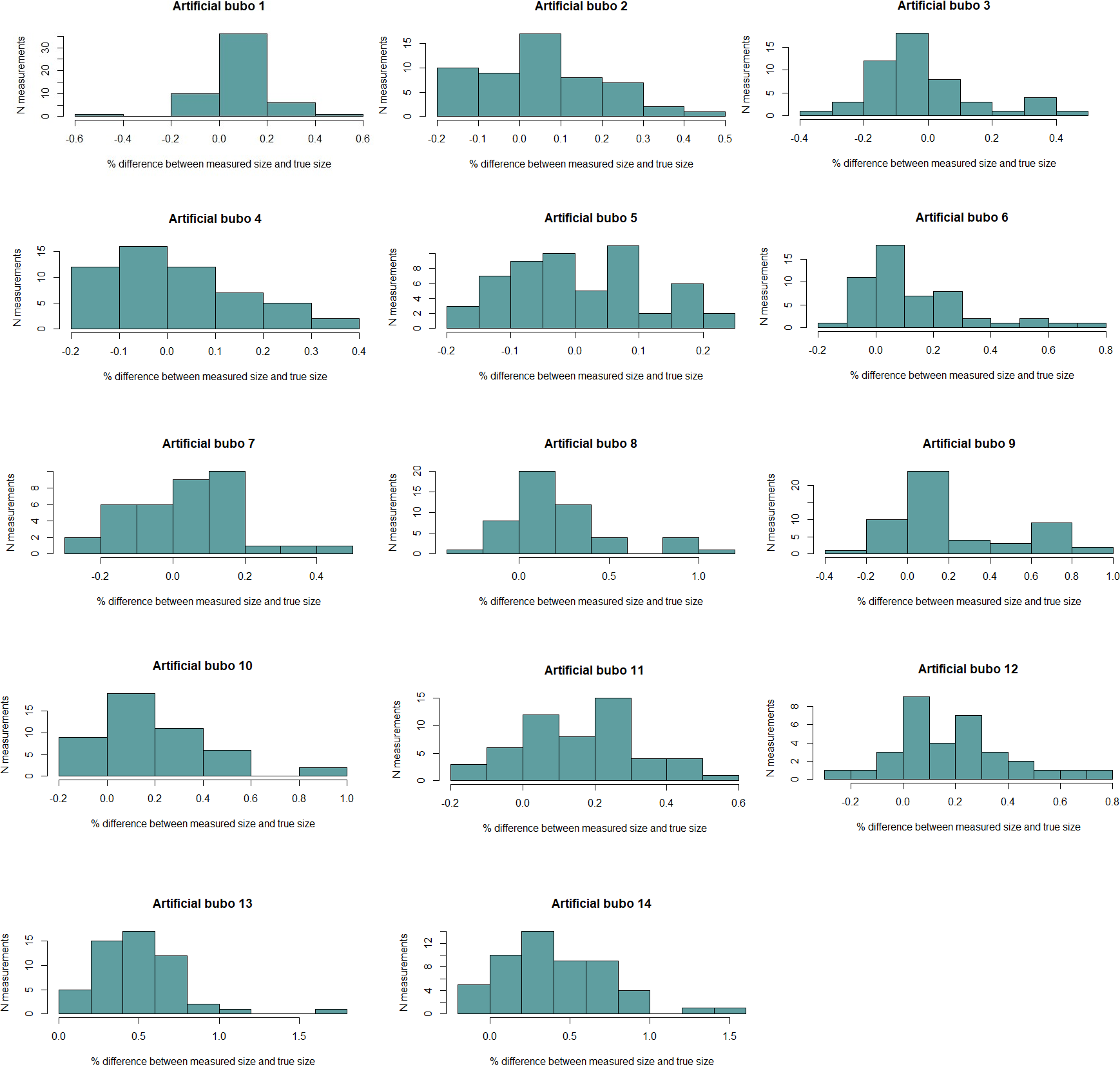

Supplement: Supplementary file 3 — Additional file 3: S3 Fig. Histograms showing the distribution of measurements in percentage difference from the true size of each artificial bubo. [file 13063_2023_7835_MOESM3_ESM.tif]

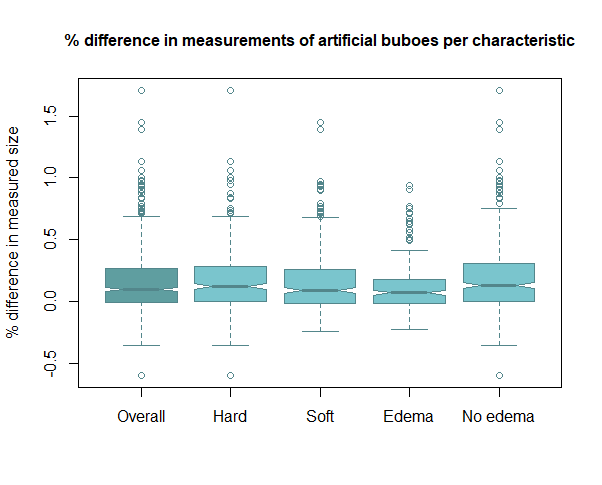

Supplement: Supplementary file 4 — Additional file 4: S4 Fig. Box plot showing the percentage difference between the true size of the artificial bubo and the measured size per characteristic. [file 13063_2023_7835_MOESM4_ESM.tiff]
