# Supplementary material for: Bubonic plague: can the size of buboes be accurately and consistently measured with a digital calliper?
Source: Trials. 2023 Dec 19;24:815. doi: 10.1186/s13063-023-07835-7 (PMC10729355; doi:10.1186/s13063-023-07835-7)
Supplement: Supplementary file 5 — Additional file 5: S5 Table. Summary of measurements. [file 13063_2023_7835_MOESM5_ESM.pdf]

**S5 Table**

**A) Linear mixed effects regression model with a random effect of rater ID and fixed effects for density, oedema, year and size (with 95% confidence interval) to determine the effect of artificial bubo characteristics on measurement accuracy**

| Characteristic | Regression coefficient | 95% CI | p-value |
|---|---|---|---|
| **Size*** | | | |
| **120.3mm** | -5.51 | -9.52 to -1.51 | 0.007 |
| **94.7** | 3.22 | -1.58 to 8.03 | 0.189 |
| **77.8** | 16.1 | 12.0 to 20.1 | <0.001 |
| **64.9** | -13.2 | 8.45 o 18.0 | <0.001 |
| **52.7** | 40.5 | 36.0 to 45.1 | <0.001 |
| **Density** | -1.76 | -5.01 to 1.48 | 0.287 |
| **Year** | 9.44 | 6.53 to 12.4 | <0.001 |
| **Presence of oedema** | 2.60 | -0.95 to 6.16 | 0.151 |

*Compared to largest artificial bubo (121.6mm)

**B) Number and percentage of measurements recorded within 5% intervals of the artificial bubo's true size, shown until 95% of all measurements are captured**

| Characteristic | 5% | 10% | 15% | 20% | 25% | 30% | 35% | 40% | 45% | 50% | 55% | 60% | 65% | 70% | 75% | 80% | 85% | 90% | 95% | 100% |
|---|---|---|---|---|---|---|---|---|---|---|---|---|---|---|---|---|---|---|---|---|
| Overall (N=698) | 179 (26%) | 307 (44%) | 403 (58%) | 464 (66%) | 508 (73%) | 550 (79%) | 574 (82%) | 592 (85%) | 610 (87%) | 627 (90%) | 638 (91%) | 645 (92%) | 657 (94%) | 664 (95%) | - | - | - | - | - | - |
| Hard (N=349) | 87 (25%) | 147 (42%) | 197 (56%) | 223 (64%) | 244 (70%) | 270 (77%) | 284 (81%) | 294 (84%) | 309 (89%) | 319 (91%) | 321 (92%) | 326 (93%) | 331 (95%) | - | - | - | - | - | - | - |
| Soft (N=349) | 92 (26%) | 160 (46%) | 206 (59%) | 241 (69%) | 264 (76%) | 280 (80%) | 290 (83%) | 298 (85%) | 301 (86%) | 308 (88%) | 317 (91%) | 319 (91%) | 326 (93%) | 328 (94%) | 336 (96%) | - | - | - | - | - |
| Oedema (N=213) | 66 (31%) | 111 (52%) | 148 (69%) | 166 (78%) | 181 (85%) | 188 (88%) | 192 (90%) | 193 (91%) | 194 (91%) | 196 (92%) | 197 (92%) | 200 (94%) | 205 (96%) | - | - | - | - | - | - | - |
| No oedema (N=485) | 113 (23%) | 196 (40%) | 255 (53%) | 298 (61%) | 327 (67%) | 362 (75%) | 382 (79%) | 399 (82%) | 416 (86%) | 431 (89%) | 441 (91%) | 445 (92%) | 452 (93%) | 458 (94%) | 466 (96%) | - | - | - | - | - |
| Size = 121.6 (N=108) | 36 (33%) | 64 (59%) | 86 (80%) | 91 (84%) | 97 (90%) | 103 (95%) | - | - | - | - | - | - | - | - | - | - | - | - | - | - |
| Size = 120.3 (N=160) | 62 (39%) | 96 (60%) | 130 (81%) | 141 (88%) | 150 (94%) | 153 (96%) | - | - | - | - | - | - | - | - | - | - | - | - | - | - |
| Size = 94.7 (N=88) | 26 (30%) | 48 (55%) | 57 (65%) | 73 (83%) | 76 (86%) | 79 (90%) | 81 (92%) | 82 (93%) | 84 (95%) | - | - | - | - | - | - | - | - | - | - | - |
| Size = 77.8 (N=150) | 32 (21%) | 56 (37%) | 78 (52%) | 91 (61%) | 101 (67%) | 112 (75%) | 114 (76%) | 120 (80%) | 122 (81%) | 127 (85%) | 130 (87%) | 132 (88%) | 137 (91%) | 137 (91%) | 140 (93%) | 141 (94%) | 142 (95%) | - | - | - |
| Size = 64.9 (N=86) | 16 (19%) | 33 (38%) | 39 (45%) | 46 (53%) | 56 (65%) | 69 (80%) | 75 (87%) | 77 (90%) | 81 (94%) | 82 (95%) | - | - | - | - | - | - | - | - | - | - |
| Size = 52.7 (N=106) | 7 (7%) | 10 (9%) | 13 (12%) | 22 (21%) | 28 (26%) | 34 (32%) | 40 (38%) | 49 (46%) | 59 (56%) | 67 (63%) | 74 (70%) | 75 (71%) | 82 (77%) | 87 (82%) | 94 (89%) | 96 (91%) | 97 (92%) | 100 (94%) | 100 (94%) | 102 (96%) |

**C) Mean absolute difference (mm), standard deviation (SD) and limits of agreement (LoA) between raters' first and second measurements for all artificial buboes and per characteristic**

| | Median percentage difference (range) | Median absolute difference, mm (range) | LoA for BA plots* | |
|---|---|---|---|---|
| | | | Lower | Upper |
| **Overall (N = 239)** | 11% (0% to 129%) | 11.84 (0.03 to 76.87) | -51.56 | 34.27 |
| **Hard (N = 121)** | 12% (0% to 104%) | 11.84 (0.14 to 64.18) | -46.84 | 31.89 |
| **Soft (N = 118)** | 11% (0% to 129%) | 12.2 (0.03 to 76.87) | -56.17 | 36.49 |
| **Oedema (N = 66)** | 12% (0% to 129%) | 12.67 (0.03 to 76.87) | -54.83 | 31.60 |
| **No oedema (N = 173)** | 12% (0% to 104%) | 11.46 (0.08 to 72.06) | -50.22 | 35.20 |
| **Size = 121.6 (N = 40)** | 9% (0% to 51%) | 11.69 (0.17 to 53.9) | -43.27 | 27.71 |
| **Size = 120.3 (N = 58)** | 9% (0% to 69%) | 11.22 (0.03 to 72.06) | -48.09 | 34.61 |
| **Size = 94.7 (N = 26)** | 10% (0% to 54%) | 10.75 (0.40 to 56.58) | -50.01 | 34.39 |
| **Size = 77.8 (N = 51)** | 15% (2% to 128%) | 13.24 (1.56 to 76.87) | -72.12 | 42.05 |
| **Size = 64.9 (N = 25)** | 16% (0% to 38%) | 1.48 (0.08 to 26.40) | -31.28 | 22.77 |
| **Size = 52.7 (N = 39)** | 12% (0% to 104%) | 10.87 (0.17 to 64.18) | -45.64 | 30.89 |

*using +ve and -ve values

**D) Average n of intrarater second measurements within 5% intervals of the rater's first measurement**

| Characteristic and average number of measurements per rater | 5% | 10% | 15% | 20% | 25% | 30% | 35% | 40% | 45% | 50% | 55% | 60% | 65% | 70% | 75% | 80% |
|---|---|---|---|---|---|---|---|---|---|---|---|---|---|---|---|---|
| **Overall (N=12)** | 3 | 5 | 7 | 8 | 9 | 10 | 10 | 11 | 11 | 11 | 11 | 11 | 11 | 12 | - | - |
| **Hard (N=6)** | 1 | 3 | 4 | 4 | 5 | 5 | 5 | 6 | - | - | - | - | - | - | - | - |
| **Soft (N=6)** | 1 | 3 | 4 | 4 | 4 | 5 | 5 | 5 | 5 | 5 | 5 | 5 | 5 | 6 | - | - |
| **Oedema (N=3)** | 1 | 1 | 2 | 2 | 2 | 3 | - | - | - | - | - | - | - | - | - | - |
| **No oedema (N=9)** | 2 | 4 | 5 | 6 | 6 | 7 | 7 | 8 | 8 | 8 | 8 | 8 | 9 | - | - | - |
| **Size = 121.6 (N=2)** | 1 | 1 | 2 | - | - | - | - | - | - | - | - | - | - | - | - | - |
| **Size = 120.3 (N = 3)** | 1 | 2 | 2 | 2 | 2 | 3 | - | - | - | - | - | - | - | - | - | - |
| **Size = 94.7 (N = 1)** | 0 | 1 | - | - | - | - | - | - | - | - | - | - | - | - | - | - |
| **Size = 77.8 (N = 2)** | 0 | 1 | 1 | 1 | 2 | - | - | - | - | - | - | - | - | - | - | - |
| **Size = 64.9 (N = 1)** | 0 | 0 | 1 | - | - | - | - | - | - | - | - | - | - | - | - | - |
| **Size = 52.7 (N = 2)** | 0 | 1 | 1 | 1 | 1 | 1 | 1 | 2 | - | - | - | - | - | - | - | - |

**E) Number and percentage of raters' second measurements recorded within 5% intervals of the raters' first measurement of the same artificial bubo, shown until 95% of all measurements are captured**

| Characteristic | 5% n (%) | 10% n (%) | 15% n (%) | 20% n (%) | 25% n (%) | 30% n (%) | 35% n (%) | 40% n (%) | 45% n (%) | 50% n (%) | 55% n (%) | 60% n (%) | 65% n (%) | 70% n (%) | 75% n (%) | 80% n (%) | 85% n (%) |
|---|---|---|---|---|---|---|---|---|---|---|---|---|---|---|---|---|---|
| Overall (N=238) | 56 (23%) | 107 (45%) | 145 (61%) | 165 (69%) | 174 (73%) | 195 (82%) | 203 (85%) | 211 (88%) | 219 (92%) | 225 (94%) | 227 (95%) | - | - | - | - | - | - |
| Hard (119) | 33 (28%) | 56 (47%) | 75 (63%) | 91 (76%) | 94 (79%) | 102 (86%) | 105 (88%) | 109 (92%) | 113 (95%) | - | - | - | - | - | - | - | - |
| Soft (N=118) | 29 (25%) | 55 (47%) | 73 (62%) | 79 (67%) | 84 (71%) | 97 (82%) | 100 (85%) | 103 (87%) | 104 (88%) | 106 (90%) | 108 (92%) | 109 (92%) | 109 (92%) | 113 (95%) | - | - | - |
| Oedema (N=65) | 10 (15%) | 28 (43%) | 44 (68%) | 47 (72%) | 49 (75%) | 56 (86%) | 58 (89%) | 58 (89%) | 58 (89%) | 59 (91%) | 61 (94%) | 62 (95%) | - | - | - | - | - |
| No oedema (N=153) | 44 (29%) | 73 (48%) | 92 (60%) | 109 (71%) | 114 (75%) | 128 (84%) | 132 (86%) | 138 (90%) | 142 (93%) | 144 (94%) | 147 (96%) | - | - | - | - | - | - |
| Size = 121.6 (N=40) | 11 (28%) | 22 (55%) | 30 (75%) | 33 (83%) | 36 (90%) | 37 (93%) | 38 (95%) | - | - | - | - | - | - | - | - | - | - |
| Size = 120.3 (N=58) | 15 (26%) | 31 (53%) | 43 (74%) | 47 (81%) | 48 (83%) | 51 (88%) | 52 (90%) | 54 (93%) | 56 (97%) | - | - | - | - | - | - | - | - |
| Size = 94.7 (N=26) | 7 (27%) | 13 (50%) | 16 (62%) | 19 (73%) | 19 (73%) | 23 (88%) | 24 (92%) | 24 (92%) | 24 (92%) | 24 (92%) | 26 (100%) | - | - | - | - | - | - |
| Size = 77.8 (N=50) | 8 (16%) | 15 (30%) | 24 (48%) | 29 (58%) | 31 (62%) | 35 (70%) | 37 (74%) | 37 (74%) | 38 (76%) | 40 (80%) | 42 (84%) | 43 (86%) | 43 (86%) | 46 (92%) | 47 (94%) | 47 (94%) | 48 (96%) |
| Size = 64.9 (N=25) | 6 (24%) | 9 (36%) | 12 (48%) | 16 (64%) | 17 (68%) | 22 (88%) | 23 (92%) | 25 (100%) | - | - | - | - | - | - | - | - | - |
| Size = 52.7 (N=39) | 9 (23%) | 17 (44%) | 20 (51%) | 21 (54%) | 23 (59%) | 27 (69%) | 29 (74%) | 33 (85%) | 33 (85%) | 34 (87%) | 35 (90%) | 36 (92%) | 37 (95%) | - | - | - | - |

**F) Linear regression using a mixed effects model (with 95% confidence interval) to determine the effect of artificial bubo characteristics on the percentage difference between the raters' first and second measurements**

| Characteristic | Estimate | 95% CI | p-value |
|---|---|---|---|
| **Size** | -0.0012943 | -0.001412802 to -0.001175798 | 0.033330 |
| **Density** | 0.0119047 | -0.05416122 to 0.07797062 | 0.724270 |
| **Presence of oedema** | 0.0493535 | -0.01926767 to 0.1179747 | 0.159961 |

**G) Number and percentage of interrater measurements that were within 5% intervals of the measurements of other raters of the same artificial bubo, shown until 95% of all measurements are captured**

| | **5% n (%)** | **10% n (%)** | **15% n (%)** | **20% n (%)** | **25% n (%)** | **30% n (%)** | **35% n (%)** | **40% n (%)** | **45% n (%)** | **50% n (%)** | **55% n (%)** | **60% n (%)** | **65% n (%)** |
|---|---|---|---|---|---|---|---|---|---|---|---|---|---|
| **Overall (N=34655)** | 6924 (20%) | 13056 (38%) | 18572 (54%) | 22903 (66%) | 26087 (75%) | 28426 (82%) | 30205 (87%) | 31294 (90%) | 32010 (92%) | 32491 (94%) | 32771 (95%) | - | - |
| **Hard (N=17286)** | 3766 (22%) | 7042 (41%) | 9867 (57%) | 12003 (69%) | 13478 (78%) | 14557 (84%) | 15363 (89%) | 15877 (92%) | 16173 (94%) | 16375 (95%) | - | - | - |
| **Soft (N=17369)** | 3158 (18%) | 6014 (35%) | 8705 (50%) | 10900 (63%) | 12609 (73%) | 13869 (80%) | 14842 (85%) | 15417 (89%) | 15837 (91%) | 16116 (93%) | 16334 (94%) | 16515 (95%) | - |
| **Oedema (N=11132)** | 2252 (20%) | 4245 (38%) | 6019 (54%) | 7415 (67%) | 8429 (76%) | 9130 (82%) | 9690 (87%) | 10034 (90%) | 10252 (92%) | 10386 (93%) | 10482 (94%) | 10587 (95%) | - |
| **No oedema (N=23523)** | 4672 (20%) | 8812 (37%) | 12553 (53%) | 15488 (65%) | 17658 (75%) | 19296 (82%) | 20515 (87%) | 21260 (90%) | 21758 (92%) | 22105 (94%) | 22289 (95%) | - | - |
| **121.6 (N=5724)** | 1477 (26%) | 2733 (48%) | 3765 (66%) | 4273 (75%) | 4930 (86%) | 5208 (91%) | 5389 (94%) | 5469 (96%) | - | - | - | - | - |
| **120.3 (N=8327)** | 1939 (23%) | 3635 (44%) | 5131 (62%) | 6219 (75%) | 6954 (84%) | 7475 (90%) | 7835 (94%) | 7930 (95%) | - | - | - | - | - |
| **94.7 (N=3912)** | 830 (21%) | 1565 (40%) | 2215 (57%) | 2696 (69%) | 3066 (78%) | 3309 (85%) | 3512 (90%) | 3650 (93%) | 3720 (95%) | - | - | - | - |
| **77.8 (N=7368)** | 1138 (15%) | 2206 (30%) | 3166 (43%) | 3963 (54%) | 4592 (62%) | 5128 (70%) | 5604 (76%) | 5999 (81%) | 6333 (86%) | 6561 (89%) | 6765 (92%) | 6904 (94%) | 6968 (95%) |
| **64.9 (N=3812)** | 700 (18%) | 1360 (36%) | 1952 (51%) | 2498 (66%) | 2935 (77%) | 3230 (85%) | 348 (90%) | 3569 (94%) | 3605 (95%) | - | - | - | - |
| **52.7 (N=5512)** | 840 (15%) | 1558 (28%) | 2343 (43%) | 3054 (55%) | 3610 (65%) | 4076 (74%) | 4417 (80%) | 4677 (85%) | 4893 (89%) | 5054 (92%) | 5119 (93%) | 5175 (94%) | 5224 (95%) |
