# Supplementary material for: Bubonic plague: can the size of buboes be accurately and consistently measured with a digital calliper?
Source: Trials. 2023 Dec 19;24:815. doi: 10.1186/s13063-023-07835-7 (PMC10729355; doi:10.1186/s13063-023-07835-7)
Supplement: Supplementary file 6 — Additional file 6. GRAAS checklist. [file 13063_2023_7835_MOESM6_ESM.docx]

| **Title and abstract** | Identify in title or abstract that interrater/intrarater reliability or agreement was investigated | COMPLETE |
| --- | --- | --- |
| **Introduction** | Name and describe the diagnostic or measurement device of interest explicitly | COMPLETE |
|  | Specify the subject population of interest | COMPLETE |
|  | Specify the rater population of interest | COMPLETE |
|  | Describe what is already known about reliability and agreement and provide a rationale for the study | COMPLETE |
| **Methods** | Explain how the sample size was chosen. State the determined number of raters, subjects/objects, and replicate observations | COMPLETE |
|  | Describe the sampling method | COMPLETE |
|  | Describe the measurement/rating process (e.g. time interval between repeated measurements, availability of clinical information, blinding) | COMPLETE |
|  | State whether measurements/ ratings were conducted independently | COMPLETE |
|  | Describe the statistical analysis | COMPLETE |
| **Results** | State the actual number of raters and subjects/objects which were included and the number of replicate observations which were conducted | COMPLETE |
|  | Describe the sample characteristics of raters and subjects (e.g. training, experience) | COMPLETE |
|  | Report estimates of reliability and agreement including measures of statistical uncertainty | COMPLETE |
| **Discussion** | Discuss the practical relevance of results | COMPLETE |
| **Auxiliary material** | Provide detailed results if possible (e.g. online) | COMPLETE |
